# The effectiveness of training on daily progress note writing by medical interns

**DOI:** 10.30476/jamp.2021.88747.1357

**Published:** 2021-07

**Authors:** IRANDOKHT SHENAVAR MASOOLEH, ELHAM RAMEZANZADEH, MARYAM YASERI, SEYYEDE SAHERE MORTAZAVI KHATIBANI, HANIEH SADAT FAYAZI, HEYDAR ALI BALOU, HOURVASH EBRAHIMI LOUYEH, FATEMEH ZAERSABET, HOSSEIN KHOSHRANG, IDEH DADGARAN

**Affiliations:** 1 Medical Education Research Center, Educational Development Center, Guilan University of Medical Sciences, Rasht, Iran; 2 Razi Clinical Research Development Centre, Guilan University of Medical Sciences, Rasht, Iran; 3 Gastrointestinal and Liver Diseases Research Center, Guilan University of Medical Sciences, Rasht, Iran

**Keywords:** Progress, Medical interns, Training, Curriculum, Hospitals, Writing

## Abstract

**Introduction:**

There is no formal education or training course about daily progress note writing in Iranian medical interns' curriculum. The current study aimed to assess the effectiveness of a training intervention on daily progress note writing by Iranian medical interns.

**Methods:**

This quasi-experimental study (pre- and post-test) was conducted on 150 medical interns selected through the census method at Razi Hospital of Rasht, north of Iran from October-2018 to May-2019. In the baseline, daily progress notes written by 150 medical interns were assessed using a Subjective, Objective, Assessment, and Plan (SOAP)-based questionnaire by the expert panel. Content validity of the questionnaire was confirmed by experts and internal consistency was determined using Cronbach's alpha coefficient. In the intervention phase, training sessions (4 one-hour sessions) on how to write the daily progress note, based on SOAP format and its importance, were held for the interns. All medical interns were given a week to meet the SOAP standards. Then, the same expert panel reviewed and assessed the newly written daily progress notes of the same medical interns. Finally, the scores from the evaluation of progress note writing, before and after the intervention, were compared with paired sample t-test.

**Results:**

The mean age of the medical interns was 23.1±5.2 years. The majority of them were male (56%). There was a significant improvement in all SOAP notes’ components written by medical interns between the pre- and post-intervention periods (general rules: 52.7±24.5 vs. 85.4±18.2, P<0.001; subjective: 21.2±18.3 vs. 61.7±24.3, P<0.001; objective: 25.3±18.3 vs. 71.3±25.2, P<0.001; assessment: 10.7±13.0 vs. 51.4±29.6, P<0.001; plan: 11.2±15.2 vs. 49.6±27.5, P<0.001; total: 21.9±13.0 vs. 61.8±23.0, P<0.001). But the scores were still far from the desirable level after the training intervention.

**Conclusion:**

The finding of the present research suggests that a training intervention can lead to some improvements in the daily progress notes written by Iranian medical interns.

## Introduction

Medical records have various uses such as follow-up care, training, research, communicating with the staff involved in health care, providing information to organizations, planning health services, improving quality of health service, supporting the patients, supporting healthcare providers, as well as evaluating the health service delivery ( 1). Medical records can help healthcare providers in planning and evaluating the patients’ treatment process and ensuring the continuity and quality of patient’s care by different healthcare providers. Medical records document the patient's condition, length of stay, examinations, treatments, clinical progress, and interactions between patient and healthcare providers during a course of treatment ( 2).

One of the most important documents in medical records is the daily progress note, which presents the patient’s latest information and daily clinical progress as well as summarizing the ongoing evaluation done by the healthcare provider team and their plan and schedule for the patient. The daily progress notes should reflect what has been diagnosed over the past 24 hours and include new patient-reported content, new points obtained from inpatient examination and paraclinical investigations, as well as differential diagnosis and planning used to write daily progress notes in the Subjective, Objective, Assessment, and Plan (SOAP) note format. The SOAP note is an acronym representing a widely used method of documentation for healthcare providers. The SOAP note is a means for healthcare providers to document in a structured and organized way ( 3). 

There is a close relationship between the clinical progress of an illness and the physician's daily orders. Thus, recording the progress of an illness along with the orders issued by the physician based on the SOAP model can increase the level of information in the records that leads to correct diagnostic and therapeutic order and enhances the educational quality of the learners. Given the need for standard documentation, health care providers need to know the correct standardized process of medical recording. Previous research reports that healthcare providers have performed poorly in completing the progress note ( 4). The results of numerous studies also demonstrate that some factors such as knowledge, attitude, training, guidelines, standards, and supervision can influence the quality of documentation and progress note writing ( 5). 

It is mentioned that one of the most useful ways to improve the quality of documents is to train medical staff to follow the principles of documentation ( 6, 7). Appropriate clinical training methods are needed to develop appropriate skills and knowledge in medical interns. Writing a progress note is a common and important component of the medical education curriculum in most countries, and its evaluation and standardization can improve the quality of medical education ( 8, 9). The progress note can be offered as an important tool to reduce medical errors and improve overall standards of patient care.

The Ministry of Health of Iran has designed and conducted a national project on clinical education criteria and indicators in training medical universities and hospitals to present a set of clinical education standards. This project resulted in the introduction of medical clinical education standards for outpatient clinics, educational rounds, grand rounds, morning reports, and journal club, and was announced in the fall of 2009. Unfortunately, despite the great importance of daily attention to the patients’ condition, clinical examinations, laboratory test results and imaging in the diagnosis and planning treatment for patients and the necessity of writing them in medical records, there is no formal educational or training course about progress note writing in Iranian medical interns' curriculum ( 10).

In other countries, due to the diversity of experience and training among health care providers, a standard progress note form has been developed to create consistency and assure a certain level of quality in the daily progress note writing and has been revised in recent years. This form enables the user to have the basic necessary information to evaluate the patient accurately and adequately. The progress note can also serve as an outcome-based learning experience for structural evaluation and assessment of the trainee’s progress ( 8, 11).

In Iran's hospitals, in a medical record, especially in progress note writing, there are many defects and shortcomings. The findings of a study in Iran showed that assistants and interns had a moderate performance in medical recording ( 5). It is expected that after completing a training course and acquiring scientific and clinical skills, medical interns will be experts in writing medical reports and progress notes ( 7).

Due to the flaws in the writing of daily progress notes in medical records and to improve the medical interns’ ability to write a progress note, the present study aimed to survey the effectiveness of a training intervention designed to improve Iranian medical interns' progress note writing skills.

## Methods

### Study Setting

This quasi-experimental study (pre- and post-test) was conducted over an 8-month period in the internal medicine ward at Razi Hospital of Rasht (affiliated to Guilan University of Medical Sciences), northern Iran. 

### Population and Sampling

The participants consisted of all general medical interns (n=150) at Razi Hospital that were enrolled during Oct.2018-May.2019. The sampling method was the census. The age and gender of all interns were recorded.

### Inclusion and Exclusion Criteria

All Iranian medical interns (male and female) at Razi Hospital who were in the 4th year of medical courses or higher and had experience in the clinical unit were included. These interns did not go through any educational course for daily progress note writing. Exclusion criterion was lack of willingness to participate in the study.

### Procedure

In the baseline, daily progress notes written by 150 medical interns were assessed by the expert panel using a SOAP-based questionnaire. The score was recorded. Then, in the intervention phase, training sessions (4 one-hour sessions) on how to write the daily progress note based on SOAP format in the patient's medical records were held for all 150 medical interns.

After the training intervention, medical interns were given a week to meet the SOAP standards. Then, the same expert panel reviewed and assessed the newly written daily progress notes of the same medical interns for a period of 20 days (150 cases) and the scores were recorded. Finally, the scores obtained from the evaluation of progress note writing based on SOAP, before and after the intervention, were compared.

### Expert Panel

The expert panel included 4 internal medicine specialists and 1 biostatistician. 

### Training Intervention

Training sessions (4 one-hour sessions) on how to write daily progress note based on SOAP were held for 2 weeks for 150 medical interns. In these training sessions, the importance, necessity and application of daily progress note writing were presented to interns by the expert panel. All medical interns received were taught about the “best practices” and instructions about the use of the SOAP note. The detected defects in daily progress notes written by medical interns in baseline were identified based on SOAP. Then, training sessions were continued using the problem-solving method, emphasizing the necessity of adhering to the standard and correct recording based on the SOAP note. At the same time, the content was presented with slides, and defects in the written daily progress report were corrected by the interns under the guidance of the expert panel. Finally, the expert panel answered some questions from medical interns in their progress note writing.

It is noticeable that after holding training sessions and scoring all medical interns after training, one workshop was conducted to detect their errors and gaps in progress note writing after 4 training sessions.

### SOAP-based Questionnaire

The data collection tool was a questionnaire based on the SOAP guidelines for writing progress notes according to the Ministry of Health of Iran (Table 1) ( 12). This questionnaire contained 47 questions in 5 components; General rule (n=7), Subjective (n=7), Objective (n=11), Assessment (n=14), and Plan (n=8). A five-point Likert scale was used for scoring (very good=5, good=4, average=3, poor=2, very poor=1, none/Blank=0). The score of each component is converted linearly to a scale of 0-100 points, with 100 representing the best score and zero denoting the worst score.

**Table1:** Practice guidelines for writing daily progress notes based on SOAP.

General rule	
1	The date of all notes is written.
2	The time (clock) of all notes is written.
3	The position of the note recorder is mentioned.
4	All notes are legible.
5	The note recorder Seal and Signature is available (for all notes).
6	The note adheres to the general principles of SOAP.
7	All of the notes areproblem oriented in general.
**Subjective**	
1	The patient's description of his/her complaint is recorded.
2	The date of the commencement of his/her complaint is recorded.
3	If the patient cannot speak, attention is given to nonverbal communication.
4	The patient's previous performance is recorded according to his/her opinion.
5	The patient’s expectation of the treatment is considered as a goal.
6	Is the problem recorded by the patient him/herself?
7	The patient's new problem intervals have been recorded.
**Objective**	
1	The results of the physical examination are recorded.
2	Onset of abilities or improvements is recorded in the examination.
3	Onset of disabilities or impairments is recorded in the examination.
4	Is the conducted intervention recorded?
5	If done, is the time of intervention recorded?
6	If done, is the location of intervention recorded?
7	If done, is the duration of intervention recorded?
8	If done, is the type of intervention recorded?
9	Are the results of laboratory test recorded?
10	Are the results of the graphs recorded?
11	Are vital signs recorded?
**Assessment**	
1	Does the Professional Assessment combine the objective + assessment components?
2	It identifies the onset of the problem or limitation.
3	Listing of the problems is accessible.
4	Prioritization of the problems is accessible.
5	The factors causing this problem are recorded.
6	Patient’s response to the intervention (improvement/ unchanged/ worsening) is recorded.
7	An emotional response to treatment is recorded.
8	The rehabilitation prognosis (excellent / good / poor / for recovery) is recorded.
9	The necessity for continued treatment is written.
10	“No transcription (copy/paste) of laboratory test” is performed.
11	A differential diagnosis or a definitive diagnosis of the problem is made.
12	The severity of the problem is recorded.
13	Problem status is recorded (for better or worse).
14	The assessment change is recorded compared to the previous day.
**Plan**	
1	Orders have been raised about whether to continue treatment or change treatment plan.
2	A distinction between short- and long-term goals has been mentioned.
3	Summarizing for both short- and long-term goals is done.
4	The goals are recorded in a specified time frame.
5	Estimates about the likely length of time required for a complete treatment plan are given.
6	A patient or family training program is outlined.
7	Follow up is provided.
8	Other expert advice is available.

SOAP: Subjective, Objective, Assessment, and Plan

The SOAP note format was used to write the progress note as follows:

Subjective (S): A brief history that the patient gives to the physician based on the previous day's history.

Objective (O): Findings obtained by the physician on the same day of the examination and based on the results of the previous day examination. In this section, the patient's vital signs, general appearance, and all para-clinical findings should be recorded.

Assessment (A): Assessment based on subjective and objective sections lead to the differential diagnosis of that day.

Plan (P): Planning, mapping and scheduling for treatment based on any differential diagnosis.

And general rule including: eligibility, signing the written note, date and time of writing, and problem oriented note.

To determine the content validity of the questionnaire, the opinions of a 10-member expert’s panel in the field of medicine and medical education were used. Based on the collected opinions, the obtained CVI and CVR were equal to 0.9 and 0.8, respectively. 

For reliability evaluation, a Cronbach's alpha coefficient of 0.98 was obtained with internal consistency method in 30 pilot subjects, and Cronbach's alpha coefficient for all items was above 0.92. 

### Statistical Analysis

Data were analyzed using SPSS, version 16.0 (SPSS Inc., Chicago, IL). Paired t-test was used to analyze the quantitative variables. P-value less than 0.05 was considered the significant level.

### Ethical Considerations

The Institutional Review Board at Guilan University of Medical Sciences approved this study (Code: 201925851245, Oct.2018). The Declaration of Helsinki, a set of ethical principles regarding human experimentation developed for the medical community by the World Medical Association, was completely considered. The written and informed consent form was completed by 150 medical interns before participating in this study. All information about medical interns and patients was confidential. The mention of medical interns’ name and signature in the progress note did not have any consequences for them. Running these surveys did not interrupt the medical interns’ duties in the hospital. Written permission from hospital authorities and internal medicine ward heads was obtained.

## Results

A total of 150 Iranian medical interns in the internal medicine ward of Razi Hospital participated in the current study. All participating interns successfully completed the protocol. Their demographic characteristics are shown in Table 2. 

**Table 2:** Iranian medical interns’ demographic characteristics

**Age, years**	
Range	18-28
Mean±SD	23.1±5.2
**Gender**	
Male	84 (56 %)
Female	66 (44 %)
**Current class**	
4^th^ or 5^th^ year	59 (39 %)
6^th^ or 7^th^ year	91 (61 %)

In the first section on adhering to the general rules of SOAP, including seal and signature of the registrant, registrar position, date and time of writing notes, legibility, and problem oriented part, the training intervention significantly improved the scores of written progress note (52.7±24.5 vs. 85.4±18.2, P<0.001).

In the Subjective component, which included the patient’s self-report, patient’s expectation of recovery, onset recorded problem, training intervention had a significant effect on improvement (21.2±18.3 vs. 61.7±24.3, P<0.001). 

The training intervention had a positive and significant effect on the Objective component including the findings of physical examinations, vital signs recordings, X-ray findings and laboratory tests, recorded improvement or impairment, and the performed interventions (time, place, duration and type of intervention) (25.3±18.3 vs. 71.3±25.2, P<0.001).

The Assessment component was the physician's differential diagnosis, and the Plan component was the planning, mapping, and scheduling based on each differential diagnosis. Both components had very low scores before the training intervention, and they achieved higher scores after the training intervention (10.7±13 vs. 51.4±29.6, P<0.001) and (11.2±15.2 vs. 49.6±27.5, P<0.001).

There was a significant improvement in the total score of SOAP note between the pre- (21.9±13) and post-intervention (61.8±23) periods (p<0.001) (Table 3).

**Table 3:** SOAP note components scores (Mean±SD) before and after training intervention

SOAP note components	Score (0-100)		Percent changes (%)	Sig.*
	Before	After		
General rules	52.7±24.5	85.4±18.2	61.7	<0.001
Subjective	21.2±18.3	61.7±24.3	190	<0.001
Objective	25.3±18.3	71.3±25.2	182.3	<0.001
Assessment	10.7±13	51.4±29.6	379.5	<0.001
Plan	11.2±15.2	49.6±27.5	342.5	<0.001
Total	21.9±13	61.8±23	182.3	<0.001

* Paired t test

SOAP: Subjective, Objective, Assessment, and Plan

## Discussion

The current findings indicate a significant improvement in daily progress note writing adherence to SOAP by Iranian medical interns after the training intervention, which is consistent with previous research. Khoshbaten et al. reviewed 1340 medical records related to internal medicine, general surgery, obstetrics, and gynaecology wards before and after the workshop and found improvement in medical recordings by medical faculty and interns ( 13).

Although there was a significant improvement in progress note writing after the training intervention, there is still considerable concern about reaching the desired level in progress notes. In the medical internship curriculum in Iran, there is no distinct course for learning progress note writing ( 10), so it is clear that a short training session may not be sufficient. Also, the lack of awareness, belief in the importance of the information recorded in the progress note, and its use in the process of evaluation and treatment lead to inadequate and incomplete writing of progress note components by medical interns. This training should be regular and continuous have importance in the curriculum, and given more attention in the quality of education of medical interns. 

The results of a study showed that adding a new "Brief Disease Progress Column" to the physician's order sheet substantially leads to recording the illness progress and causes of the given medical orders based on the SOAP note ( 4). However, in the present study, a training intervention was done to improve the progress note writing based on SOAP. 

The results of this study indicate that there were many defects in progress note writing by medical interns at baseline. Mashoufi et al. mentioned the poor and incomplete writing of medical records by physicians in the main groups of health care providers ( 14). The study by Seyf Rabiei et al. also confirms that there are serious deficiencies in hospital records ( 15). Without proper documentation of medical records and progress notes, it can be difficult to prove that health services provided to the patient were acceptable or necessary. Inadequate or incomplete records have numerous consequences, and the patient is the first person to experience these consequences ( 16).

Azimi et al. found that none of the 1800 medical records was fully compatible with standards, and 70% of the records had at least one error recorded by physicians ( 17). In the present study, there was a significant improvement in the recording of progress standards before and after the training program; more standards were met. The results of the study by Kimiafar et al. showed that documenting medical records is performed incompletely by care providers, leading to the loss of necessary information for providing optimum health care ( 2). Accurate writing of hospital progress notes is essential and, if neglected, can slow the research in hospital settings and has legal implications for the medical team. In addition, incomplete documentation can lead to insurance deductions and financial consequences, which have been cited in various studies ( 6, 13)

Seo et al. examined 95 SOAP notes recorded by Korean medical interns at five universities, showing that 36.8% were unsigned. Only 27.4% recorded the patient's symptoms as the Objective component; however, all interns correctly recorded the Subjective component, and accurate recordings of symptoms, physical examination, diagnoses, and planning were 78.9 %, 9.5 %, 62.1 %, 0.38 %, respectively. In sum, the SOAP notes recorded by Korean medical interns were not complete, appropriate, or accurate, and the researchers suggested that a review and training program should be presented to medical interns ( 18). In the present study, there was a significant improvement in the status of physician data entry (patient name, date and time, physician seal and signature) in the progress note after the SOAP training intervention. The results of Farokhi et al.'s study showed that the training workshop had positive effects on medical interns' recoding ( 19).

There are several reasons for medical interns' failure to adhere to the correct principles of progress note writing, such as overcrowding of the ward, lack of responsibility, absence of a reward and punishment system, and most importantly, insufficient training ( 15, 20). Kahouei et al. also stated training of medical interns and ongoing monitoring of the documentation of health care provided to patients improved the process of documenting medical records ( 21).

Several studies have shown that there are still deficiencies in progress note writing based on SOAP standards in Iranian medical universities and hospitals that require serious training interventions ( 13- 16). The results of this study showed a significant improvement in adherence to the SOAP-based standard practice of progress note writing, which demonstrates the effectiveness of training although there is still a gap to the desirable level. It shows that a variety of continuous training methods such as workshops, classrooms, and e-learning combinations can be used to reach the best outcome. Speech is one of the traditional ways of teaching; despite the development of new and innovative teaching methods such as problem-based learning and increasing use of computers and the Internet, evidence shows that using good content and lecturer can produce positive, reasoned, and appropriate outcomes from training. But using questions in the learning process is a better way because it leads to developing critical thinking and problem-solving skills in learners. In addition, this method provides an opportunity for the teacher to observe and listen to the learner. Therefore, several teaching methods that can improve the learning process ( 13, 19) were used simultaneously in the current study. In this regard, Kiviniem also concludes that integrated teaching methods can be an effective tool for optimizing the interns' education and improving their performance in medical sciences ( 22). Management in the training and implementation of the standardization program through continuous training, active supervision and feedback have a significant impact on improving medical interns’ progress note writing that requires knowledge, responsibility, and time spent by the faculty as well as educational experts.

In disagreement with the current findings, Storjohann et al. did not find a significant difference between the mean scores of SOAP notes recorded by interns after attending a SOAP note workshop. The interns concluded that attending a workshop could be helpful for their learning ( 23).

### Limitations of study

Despite the practical finding of the present study that could be useful for improving and enhancing intern progress note writing, this study had some limitations. One of the limitations is that the present study was conducted solely on the medical records of one hospital, so generalization of finding to all hospitals in Iran should be done with caution. The lack of a control group for precise control of some of the confounding variables was another limitation of the present study. The researchers attempted to control it by identifying some of the known confounding variables, such as receiving recent training in progress note writing, which were excluded from study.

## Conclusion

The findings of the current study demonstrated improvement in daily progress note writing, and adherence to its standard principles based on SOAP note format by Iranian medical interns after SOAP method training intervention.

## Funding

A research grant was received from Guilan University of Medical Sciences to carry out this project.

## Ethics approval and consent to participate

The Institutional Review Board at the Guilan University of Medical Sciences approved this study (Code: 201925851245, Oct.2018). The Declaration of Helsinki, a set of ethical principles regarding human experimentation developed for the medical community by the World Medical Association, was completely respected. The written and informed consent form was completed by 150 medical interns before participating in this study. All information about medical interns and patients was confidential. The mention of medical interns’ name and signature in the progress note did not have any consequences for them. Running these surveys did not interrupt medical interns’ duties in the hospital. Written permission from hospital authorities and internal medicine ward heads was obtained.
